# Evaluation of the Visually Impaired Experience of the Sound Environment in Urban Spaces

**DOI:** 10.3389/fpsyg.2021.731693

**Published:** 2022-01-05

**Authors:** Sen Zhang, Ke Zhang, Meng Zhang, Xiaoyang Liu

**Affiliations:** ^1^School of Architecture, Tianjin University, Tianjin, China; ^2^School of Urban and Rural Construction, Hebei Agriculture University, Baoding, China; ^3^School of Urban Design, Wuhan University, Wuhan, China

**Keywords:** visually impaired people, sound environment evaluation, urban design, urban space, independent sample non-parametric test

## Abstract

Visually impaired people have unique perceptions of and usage requirements for various urban spaces. Therefore, understanding these perceptions can help create reasonable layouts and construct urban infrastructure. This study recruited 26 visually impaired volunteers to evaluate 24 sound environments regarding clarity, comfort, safety, vitality, and depression. This data was collected in seven different types of urban spaces. An independent sample non-parametric test was used to determine the significance of the differences between environmental evaluation results for each evaluation dimension and to summarize the compositions of sound and space elements in the positive and negative influence spaces. The results suggested that visually impaired people (1) feel comfort, safety, and clarity in parks, residential communities, and shopping streets; (2) have negative perceptions of vegetable markets, bus stops, hospitals, and urban departments; (3) feel anxious when traffic sounds, horn sounds, manhole cover sounds, and construction sounds occur; and (4) prefer spaces away from traffic, with fewer and slower vehicles, with a suitable space scale, and moderate crowd density. These results provide a reference for the future design of activity venues (i.e., residential communities, vegetable markets, bus stops, parks, shopping streets, hospitals, and urban functional departments) and the planning of accessibility systems for visually impaired urban residents.

## Introduction

According to several surveys, including the second sampling survey of Chinese people with disabilities, there were approximately 85 million disabled people in China in 2018, with the total number predicted to be nearly 100 million by the end of 2021 (Kang et al., 2019). In addition, the World Health Organization survey report indicated that, among people with disabilities in China, approximately 17 million are visually impaired, accounting for 20% of the global total and making China home to the largest number of visually impaired people in the world, most of whom live in its cities (Jianghua and Juhui, 2012). Visually impaired people suffer from varying degrees of visual impairments. To illustrate, they cannot obtain information about the external environment through vision and their ability to interact with space is extremely limited. Some research has shown that vision is the main perceptual channel from which humans access external information. Furthermore, information acquired through vision accounts for 83% of the total information processed by humans. If visual problems arise, human perception of the spatial environment is almost completely lost (Treichler and Agee, 1983). However, visually impaired people can feel the characteristics of various urban spaces through the sound environment. Therefore, through visually impaired people’s perception and evaluation of the sound environment, we can understand their needs for the use of different spaces in a city. This is important to optimize the urban spatial layout and improve the construction of urban barrier-free service facilities.

The study of the behavior of visually impaired peoples began in the early twentieth century. From 1985 to 2000, Shields (1994), Dunlop et al. (1996), Boyce et al. (1999), and other researchers studied the evacuation speed among people with disabilities (visually/hearing/motion-impaired people) in buildings with different functions. They showed that the walking speed of visually impaired and hearing-impaired people was not much slower than that of non-disabled people under the same conditions. Moreover, Pearson and Joost (1983) investigated the evacuation speed in the living environments of the visually impaired, elderly, and wheelchair-bound disabled people. Clark-Carter et al. (1986) investigated the relationship between the complexity of the external environment and the evacuation speed of visually impaired people. In addition, Robertson and Dunne (1998) conducted experiments in buildings to compare the effects of spatial guidance system designs on the activities of visually impaired people in external environments. Sørensen and Dederichs (2015) found that the walking speed of visually impaired people was the same as the walking speed of non-disabled adults and was not affected by density on the stairs. However, other studies have shown that the average free walking speed of visually impaired people may depend on the degree of vision loss. There is sufficient research on evacuation behavior of people with a visual impairment.

Some scholars conducted research on the interaction between visually impaired people and urban spaces. For example, Bentzen et al. (2004) analyzed the mental image representation of the environment created by visually impaired people and suggested that they focus on the connection points between different roads (intersection information). To illustrate, Gaunet (2006) proposed that the information required for the travel of visually impaired people should mainly include the path information within a 5–10 m radius during walking and the environmental information within a 30 m radius of an intersection. Additionally, Koutsoklenis and Papadopoulos (2011) evaluated the olfactory experience of visually impaired people to explore factors affecting their travel in an urban environment. Furthermore, Kan-Kilic and Dogan (2017) conducted field route perception experiments for visually impaired people and concluded that the sound of the city and the echo of the environment were the most important factors for participants in dense urban environments. Campisi et al. (2021) evaluated the walkability of urban environments from the perspective of visually impaired people, analyzed the impact of street material elements on their activities, and evaluated their psychological experience of walking along a path. These studies are mainly qualitative involving observing people with a visual impairment, communicating with them, or conducting some experiments. However, due to the limited spatial scope of these studies and the single type of urban space, these conclusions have certain limitations and cannot provide reference for a larger region or other cities.

With in-depth research on visually impaired individuals, related research on urban space environmental assessments is gradually being conducted. For example, Steyvers and Kooijman (2009) conducted an environmental cognitive map experiment on visually impaired people and normal vision subjects in a virtual environment. The results suggested that the two groups showed significant differences in generating cognitive maps based on the auditory environment. Jianxi and Xinren (2020) classified the urban walking space affecting the use of visually impaired people into three sections—visually impaired people, landscape space, and urban space—and found that sound was the core factor affecting security. Thus, it is evident that sound elements have an important impact on visually impaired people’s cognition of the urban environment, which is important for relevant research in the future.

In China, there is a lack of relevant research on people with a visual impairment in the field of urban construction, with existing research focusing on improving barrier-free facilities. For example, Chuan-sheng et al. (2009) analyzed the walking speed of people with disabilities and elderly people using an experiment and proposed a barrier-free design strategy. Wen (2013) investigated the physiological, behavioral, and demand characteristics of people with a visual impairment and proposed “blind painting entertainment equipment” to meet their daily life needs. Jiang (2018) took the Xi’an subway as an example, analyzed the behavior characteristics of people with a visual impairment, and constructed a complete set of subway barrier-free guidance systems. Zhang S. et al. (2018) collected data on the walking characteristics and ability of visually impaired people by utilizing a questionnaire survey and testing walking speed, redefining the service radius of urban shelters, designing the layout of shelter and blind tracks, building a suitable multilevel evacuating system, and making some suggestions to urban safety construction. Thus, these related studies are relatively superficial. The research method is also relatively simple, mainly questionnaire surveys or interviews, and the conclusion only puts forward some conceptual suggestions. That is, the research conclusions are not sufficient to support urban construction. This is also closely related to the particularity of people with a visual impairment. They rarely participate in various social activities; thus, it is difficult to recruit volunteers and conduct research.

At present, China is in a stage of rapid urbanization; however, the urban and social structure are not stable enough. Concurrently, the main goal of urban construction should meet the living needs of all kinds of people. Therefore, further research on people with a visual impairment is of great practical significance to improve urban construction and ensure social fairness and stability. Existing research shows that the sound environment plays an important role in the perception of urban spaces for people with a visual impairment. Based on this, visually impaired people were selected for this study and asked to evaluate the sound environment of different urban spaces. According to the evaluation results, the elements of the acoustic environment and urban spaces were analyzed. By comparing evaluation differences, we determined the urban sound elements and space composition that positively impacted the visually impaired.

## Data and Methods

The volunteers with a visual impairment in this experiment were from the Tianjin Blind Association. In this study, some typical urban public spaces for audio and video recording were selected. Subsequently, visually impaired people conducted a perceptual evaluation of the acoustic environment. Through the independent sample non-parametric test based on IBM SPSS Statistics V22, from the two aspects of urban space type and equivalent sound pressure level, the urban space elements and sound elements under different evaluation dimensions were identified.

As of 2021, the number of people registered with a visual impairment in Tianjin was approximately 40,000. The scope of this study was limited to Heping District, the core urban area of Tianjin, China. The Heping District performs important urban functions containing facilities for economic, cultural, and political activities. It is also a densely populated urban area, with a permanent population of approximately 350,000. In this study, the 26 volunteers with a visual impairment were from this area and were between 30 and 65 years old. Table 1 provides basic information about the volunteers.

**TABLE 1:** Basic information describing visually impaired volunteers.

		Frequency	Percentage
Age	30–35	6	23.08%
	36–59	10	38.46%
	60–65	10	38.46%
Gender	Male	14	53.85%
	Female	12	46.15%
Employment status	Yes	15	57.69%
	No	11	42.31%
Education level	Elementary school and below	10	38.46%
	Junior and senior high schools	16	61.54%
	University and above	0	0.00%
Spouse	Live together	20	76.92%
	Live alone	6	23.08%

### Urban Public Space Selection

**FIGURE 1:**
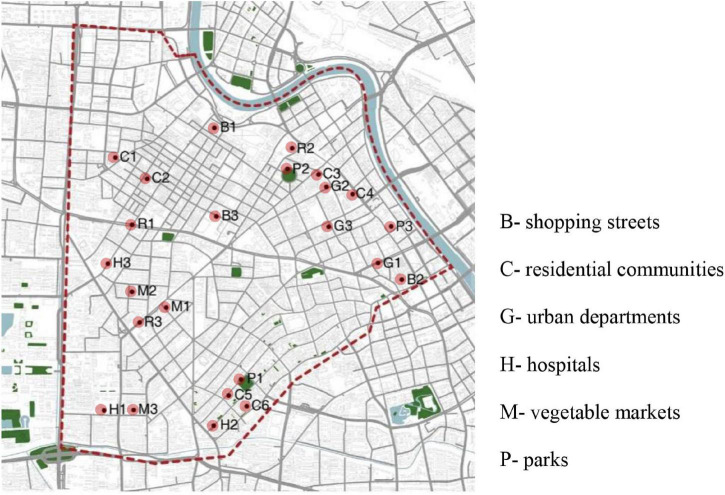
Distribution of scene recording points.

According to relevant research results (Jaeger and Bowman, 2005; Wen et al., 2014; Qianwen et al., 2016; Sen et al., 2021), seven types of urban spaces—residential communities, vegetable markets, bus stops, parks, shopping streets, hospitals, and urban functional departments—have high travel frequency. Based on these urban spaces, a total of 24 scenes were selected in this study; all scenes were outdoors. Among them, three scenes were randomly selected for each type of space, and six scenes were selected for the residential community space (as shown in Figure 1). The recording time was from March 20 to 21, 2021, 10:00 a.m.–12:00 a.m. A TES-1357 sound level meter was used for sound pressure level measurement. A SONY PCM-A10 two-channel recorder was used for environmental sound recording, and the iPhone 12 Pro Max was used for environmental video recording.

### Questionnaire Design

The perception and evaluation of urban spaces is an important research topic in urban planning, geography, environmental psychology, and other major fields (Lynch, 1960; Ulrich, 1983; Kaplan and Kaplan, 1989; Zhang F. et al., 2018). Based on the related research results of urban space evaluation, the questionnaire used in this study identified five evaluation dimensions: clarity, comfort, safety, vitality, and depression. Each dimension was evaluated on a scale from 1 to 5.

Clarity refers to the degree to which visually impaired individuals can distinguish various sound sources in a scene, with a higher score indicating higher discrimination of the sound source. Comfort refers to the degree to which visually impaired people feel relaxed and let go of their mental vigilance, with a higher score indicating a higher degree of relaxation. Comfort refers to the degree to which a visually impaired person feels relaxed and drops their mental alertness, with a higher score indicating a higher level of relaxation. Safety refers to the degree to which visually impaired individuals are threatened by the surrounding environment, with a higher score indicating a higher threat. Vitality is used to judge the attractiveness of the scene to the visually impaired, with a higher score indicating a more attractive scene. Depression refers to the degree of disgust and negative emotions of the visually impaired, with a higher score indicating a higher negative sentiment.

### Experimentation

**FIGURE 2:**
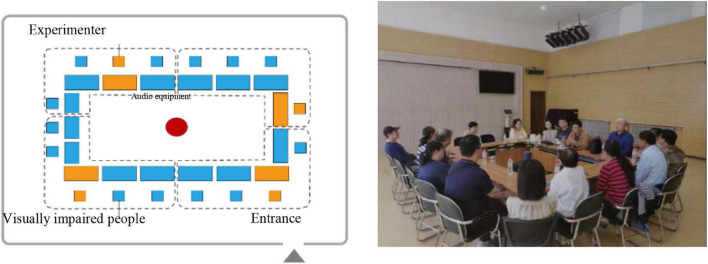
Layout and photo of experiment setup.

In a previous study, we found that volunteers with a visual impairment were unable to participate in experiments in more professional experimental settings. Therefore, the activity room of the Tianjin Blind Association was selected as the experimental site. This was also the main place for their daily activities. There were no other facilities in the activity room except for tables and chairs. The experiment used a Bose Soundlink Revolve + 360° surround sound amplifier device to play sound. During the experiment, 24 scene recordings were played, and each scene lasted 30 s. Before the start of the experiment, we measured the sound pressure level of the recording material through the sound level meter and adjusted the volume. In this way, the sound pressure level received at each seat was as close as possible to that recorded on site to ensure the accuracy of the experimental results. The experiment was conducted in two groups, with 13 visually impaired volunteers and four experimental assistants in each group (as shown in Figure 2). After each scene was played, the experimental assistant recorded the results of each person’s evaluation. All experiments lasted approximately 80 min in total.

## Results

### Scene Sound Information Statistics

According to the video and audio recording material, the sound element information of each scene was counted by the research team. A total of seven types of sounds occurred: traffic sounds, horn sounds, manhole cover sounds, chat sounds, walking sounds, hawker sounds, and construction sounds. The traffic sound was the sound of tires rubbing against the ground, the horn sound of motor vehicles and non-motorized vehicles sounding their horns, the manhole cover sound of car tires hitting the top of the manhole cover, the chat sound of conversations between passers-by within the scene, the walking sound of passers-by walking, and the hawker sound of a building or individual advertising their goods, such as a shop or supermarket. The distribution of sound information for each scene is shown in Table 2. Almost every scene contained traffic and horn sounds, and a few spaces contained sound sources such as building construction noises.

**TABLE 2:** Sound information of each scene.

	Scene number	Traffic Sound	Horn sound	Manhole cover sound	Chat sound	Walking sound	Hawker sound	Construction sound
Market	1	•		•			•	
	2	•		•			•	
	3	•		•			•	
Bus stop	4	•	•				•	
	5	•	•					•
	6	•	•				•	
Park	7				•	•		
	8	•	•		•	•		
	9	•			•			
Shopping streets	10				•	•		
	11	•			•		•	
	12				•		•	
Residential community	13	•	•		•			
	14	•	•		•			
	15	•	•					
	16	•	•					
	17	•	•	•				•
	18	•	•					
Hospital	19	•	•	•				
	20	•						
	21	•	•		•		•	
Urban department	22	•	•		•			•
	23	•	•					
	24	•	•				•	

**FIGURE 3:**
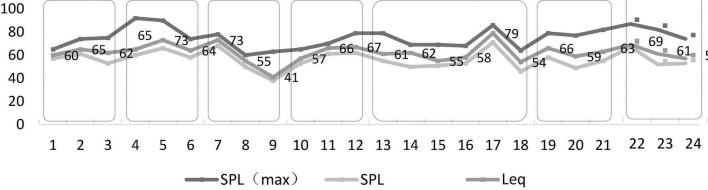
Statistical results of the sound pressure level of each scene.

Figure 3 shows the statistical results for the equivalent sound pressure levels and minimum and maximum sound pressure levels for each scene. The equivalent sound pressure levels ranged between 40 and 80 dB, with a more pronounced difference between scenes. The maximum sound pressure level in Scene 4 and Scene 5 exceeded 90 dB. The minimum sound pressure level in the park was less than 40 dB. The maximum and minimum sound pressure levels of bus stations, parks, hospitals, and government departments were quite different. Referring back to the video, there were multiple horn sounds in these scenes. This gap is closely related to road traffic conditions.

**FIGURE 4:**
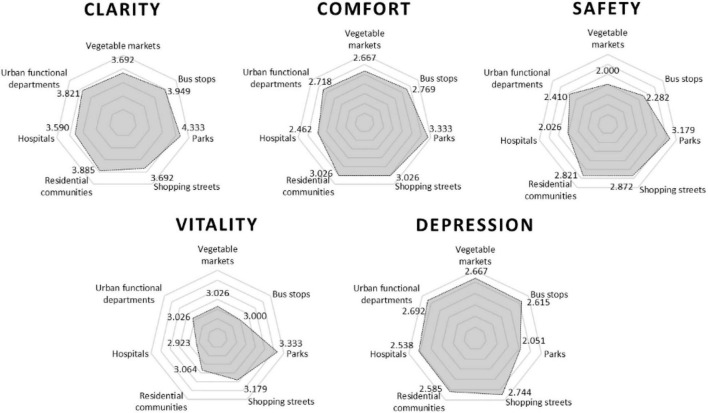
Evaluation results of each evaluation dimension.

Figure 4 shows the average score statistics for each evaluation dimension. Regarding clarity, the park had the highest average score of 4.333. Regarding comfort, parks had the highest average rating of 3.333, followed by residential community and shopping streets with 3.026. Regarding safety, parks had the highest average rating of 3.179, followed by residential community and shopping streets with 2.821 and 2.872, and vegetable markets and hospitals with lower ratings of 2.000 and 2.026. Regarding vitality, the evaluation of each scene was quite different; the highest evaluation of parks was 3.333, and the lowest evaluation of hospitals was 2.923. Regarding depression, the highest evaluation of commercial blocks was 2.744, and the lowest evaluation of park scenes was 2.051. Furthermore, when there were horn, manhole cover, and construction sounds in the scene, the visually impaired felt anxious and afraid. Some people stated that “I will not move, I can only wait for help.”

Comprehensive results of sound environment evaluation, sound type recognition, and sound pressure level statistics were obtained. Parks, residential communities, and shopping streets were better evaluated regarding safety and comfort. From the audio and video materials, it was evident that there were no traffic elements in these spaces; therefore, relevant sounds were rarely produced. Vegetable markets, bus stops, and hospital spaces had a complicated sound environment due to the large traffic volume and mixed traffic of people and vehicles. Accordingly, the equivalent and maximum sound pressure levels were higher. Visually impaired people generally had certain negative comments in these types of spaces. In the next stage of the study, the acoustic environmental conditions and urban space types were analyzed through independent sample non-parametric tests and the significance of differences in the results of each evaluation dimension were detected.

### Independent Sample Non-parametric Test

The 24 scenes were divided into two groups, and an independent sample non-parametric test was performed on each group. Group A took the urban space type as a variable, and Group B took the equivalent sound pressure level as a variable. First, the test results of Group A showed that there was no significant difference in the evaluation results of vitality and depression. This means that, under these two dimensions, there is no significant difference in the impact of the seven types of spaces on the psychological perceptions of the visually impaired. The results of the other three dimensions tested are shown in Table 3.

**TABLE 3:** Pairwise comparisons of space types.

Comfort	1	2	3	4	5	6	7
Vegetable markets(1)	–	1.00	0.00**	0.01**	0.00**	1.00	1.00
Bus stops(2)	1.00	–	0.00**	0.73	0.20	0.41	1.00
Parks(3)	0.00**	0.00**	–	0.10	0.04	0.00**	0.00**
Shopping streets(4)	0.01**	0.73	0.10	–	1.00	0.00**	0.12
Residential communities(5)	0.00**	0.20	0.04	1.00	–	0.00**	0.02*
Hospitals(6)	1.00	0.41	0.00**	0.00**	0.00**	–	1.00
Urban departments(7)	1.00	1.00	0.00**	0.12	0.02*	1.00	–
**Clarity**							
Vegetable markets(1)	–	0.15	0.00**	1.00	1.00	1.00	1.00
Bus stops(2)	0.15	–	0.02*	0.08	1.00	0.01**	1.00
Parks(3)	0.00**	0.02*	–	0.00**	0.00**	0.00**	0.00**
Shopping streets(4)	1.00	0.08	0.00**	–	0.70	1.00	1.00
Residential communities(5)	1.00	1.00	0.00**	0.70	–	0.07	1.00
Hospitals(6)	1.00	0.01**	0.00**	1.00	0.07	–	1.00
Urban departments(7)	1.00	1.00	0.00**	1.00	1.00	1.00	–
**Safety**							
Vegetable markets(1)	–	0.22	0.00**	0.00**	0.00**	1.00	0.03*
Bus stops(2)	0.22	–	0.00**	0.00**	0.00**	0.50	1.00
Parks(3)	0.00**	0.00**	–	1.00	0.11	0.00**	0.00**
Shopping streets(4)	0.00**	0.00**	1.00	–	1.00	0.00**	0.01**
Residential communities(5)	0.00**	0.00**	0.11	1.00	–	0.00**	0.01**
Hospitals(6)	1.00	0.50	0.00**	0.00**	0.00**	–	0.07
Urban departments(7)	0.03*	1.00	0.00**	0.01**	0.01**	0.07	–

**P ≤ 0.01, *P ≤ 0.05. Bold values indicate significant differences between the two data.

In the results of the comfort analysis, it was evident that the evaluation results of the park space was significantly different from the evaluation results of vegetable markets, bus stations, hospitals, and urban departments. The evaluation results of vegetable markets were significantly different from those of shopping streets and residential areas; there were also significant differences between the evaluation results of shopping streets and those of hospitals. Combined with the experimental statistics, parks, shopping streets, and residential areas scored the highest regarding comfort, which positively impacted the psychology of visually impaired people. In the results of the clarity analysis, the evaluation results of parks was significantly different from other types of urban spaces. The evaluation results of bus stations and hospitals were also significantly different. Regarding safety, the evaluation results of parks, shopping streets, and residential areas were significantly different from those of the other four types of urban spaces. Thus, the park had the best evaluation regarding clarity. Regarding safety and comfort, parks, residential communities, and shopping streets were rated the best. Therefore, parks, shopping streets, and residential areas had a positive effect on the psychology of visually impaired people. Comparatively, the other four types of spaces had negative influences.

Second, Group B was tested. According to on-site statistics, the equivalent sound pressure levels of the 24 scenes was in the range of 40–80 dB. The experiment used 5 dB as the division scale and divided scenes into seven groups. The three scenes in the park were in the range of 41–45 dB; the various scenes in the shopping streets, hospitals, and urban departments were in the range of 50–70 dB; the three scenes in the market were in the range of 60–65 dB; the three scenes in the bus stop were in the range of 60–75 dB, and the scenes in the residential community were in the range of 50–80 dB. The non-parametric test results were similar to those of Group A, showing that there was no significant difference in the evaluation results of vitality and depression. This means that, under these two dimensions, there is no difference in the effect of different levels of equivalent sound pressure levels on the psychological perception of people with a visual impairment. The results of the other three dimensions tested are shown in Table 4.

**TABLE 4:** Pairwise comparisons of equivalent sound pressure levels.

Comfort	1	2	3	4	5	6	7
41–45 dB(1)	–	0.15	0.01**	0.00**	0.00**	0.00**	0.00**
51–55 dB(2)	0.15	–	1.00	0.03*	0.00**	0.12	0.32
56–60 dB(3)	0.01**	1.00	–	0.49	0.01**	0.85	1.00
61–65 dB(4)	0.00**	0.03*	0.49	–	1.00	1.00	1.00
66–70 dB(5)	0.00**	0.00**	0.01**	1.00	–	1.00	1.00
71–75 dB(6)	0.00**	0.12	0.85	1.00	1.00	–	1.00
76–80 dB(7)	0.00**	0.32	1.00	1.00	1.00	1.00	–
**Clarity**							
41–45 dB(1)	–	0.03	0.01**	0.00**	0.00**	0.23	0.06
51–55 dB(2)	0.03	–	1.00	1.00	0.02**	1.00	1.00
56–60 dB(3)	0.01**	1.00	–	1.00	0.04*	1.00	1.00
61–65 dB(4)	0.00**	1.00	1.00	–	0.00**	1.00	1.00
66–70 dB(5)	0.00**	0.02**	0.04**	0.00**	–	0.01**	1.00
71–75 dB(6)	0.23	1.00	1.00	1.00	0.01**	–	
76–80 dB(7)	0.06	1.00	1.00	1.00	1.00	1.00	–
**Safety**							
41–45 dB(1)	–	0.39	0.01**	0.00**	0.00**	0.00**	0.00**
51–55 dB(2)	0.39	–	1.00	0.00**	0.08	0.26	0.40
56–60 dB(3)	0.01**	1.00	–	0.01**	1.00	1.00	1.00
61–65 dB(4)	0.00**	0.00**	0.01**	–	1.00	1.00	1.00
66–70 dB(5)	0.00**	0.08	1.00	1.00	–	1.00	1.00
71–75 dB(6)	0.00**	0.26	1.00	1.00	1.00	–	1.00
76–80 dB(7)	0.00**	0.40	1.00	1.00	1.00	1.00	–

**P ≤ 0.01, *P ≤ 0.05. Bold values indicate significant differences between the two data.

Regarding comfort, the 41–45 dB evaluation results were significantly different from the equivalent sound pressure levels of the other six groups. Regarding clarity, when the equivalent sound pressure level was in the range of 66–70 dB, the evaluation results were significantly different from those of the other groups. Safety analysis results were the same as the results of the comfort analysis. Moreover, 41–45 dB was significantly different compared to the other groups. When the equivalent sound pressure level of the sound environment was between 41 and 44 dB, various scenes in parks made visually impaired people feel comfortable and safe. Comparatively, when the equivalent sound pressure level of urban spaces exceeded 65 dB, such as bus stops, vegetable markets, and urban departments, they felt uncomfortable and unsafe. When the equivalent sound pressure level was 66–70 dB, visually impaired people had the highest ability to distinguish various sound sources in the environment.

Based on the results of the non-parametric test, the attribute elements of each type of space were analyzed, as shown in Table 5. Parks, residential communities, and shopping streets belonged to the types of spaces that positively impacted people with a visual impairment; this was reflected in safety, comfort, and clarity.

**TABLE 5:** Statistics of attribute elements of each type of space.

	Space type	Evaluation	Leq (dB)	Sound type	Space characteristics
	Park	Safety, comfort, clarity	41–64	Traffic sounds, chat sounds, walking sounds	Away from traffic roads, few people, few buildings
Positive	Residential	Safety, comfort	54–79	Traffic sounds, horn sounds, manhole cover sounds, construction sounds	Narrow traffic road, slow vehicles speeds, few people,
	Shopping street	Safety, comfort	57–67	Chat sounds, hawker sounds	Narrow traffic road, slow vehicle speeds, shops along the street, blind roads
	Bus stop	No safety, discomfort	64–73	Traffic sounds, horn sounds, hawker sounds, construction sounds	Close to the traffic road, many vehicles with fast speeds, many people
	Vegetable market	No safety, discomfort	60–65	Traffic sounds, manhole cover sounds, hawker sounds	Close to the traffic road, many people, many parking spaces
Negative	Hospital	No safety, lack of clarity, discomfort	59–66	Traffic sounds, horn sound, hawker sounds, construction sounds	Close to the traffic road, many people
	Urban department	No safety, discomfort	57–69	Traffic sounds, horn sound, chat sounds, hawker sounds, construction sounds	Close to the traffic road, many vehicles with fast speeds, many people, blind roads

These space types had some common characteristics. First, regarding sound characteristics, the equivalent sound pressure level was low, with some scenes (residential communities) being slightly high, generally in the range of 50–60 dB, which is positive for the psychological perception of people with a visual impairment. Second, regarding sound elements, there were a small number of hawker and manhole cover sounds, most of which were dominated by chat and hawker sounds; these were non-transportation sounds that are generally favorable to visually impaired individuals. Third, regarding space characteristics, the three types of urban spaces (parks, residential communities, and shopping streets) were all far away from urban roads or had low traffic road levels. Therefore, there were few cars in the space and the speed of cars is not fast. There were also relatively few people in the space. Moreover, shopping streets spaces also have blind roads.

Bus stops, markets, hospitals, and urban departments had a negative psychological impact on people with a visual impairment, reflected in non-safety, discomfort, and lack of clarity. First, regarding sound characteristics, the equivalent sound pressure level of each scene in these four types of spaces was high, mostly in the range of 60–70 dB. Second, regarding sound elements, these scenes included several horn sounds, manhole cover sounds, and building construction sounds, which are unfavorable to people with a visual impairment. Third, regarding space characteristics, these scenes were generally adjacent to traffic roads, with several manhole covers and many people. Thus, the volume of vehicles was large and the speed of traffic was fast. Some scenes also had many parking spaces. In these negative influence spaces, there were also some common features, such as sudden high-decibel sounds. In particular, bus stops were more prominent and belonged to the convergence point of various transportation vehicles in the city.

## Discussion

The analysis results showed that the sound pressure level indicators and traffic sounds had a significant effect on the psychological feelings of visually impaired people. They would show a relatively positive attitude when the scene was far from the traffic, or when there was little interference from traffic sounds and the sound pressure level indicator was kept within 60 dB. By comparing positive and negative scenes, people with a visual impairment preferred urban scenes that are quiet, away from traffic roads, and have moderate crowd density. Moreover, they were very sensitive to horn, construction, and manhole cover sounds, which can cause anxiety. Thus, when they are in an urban space with such characteristics, they may not be able to travel properly or make proper judgments. Therefore, when the city starts construction and plans the layout of barrier-free facilities in the future, the main places for visually impaired people to travel daily can be combined with parks, residential areas, and commercial streets and can be kept as far as away possible from busy urban roads. This will help enhance their city experience and reduce negative psychological feelings.

Compared with previous studies, this study further explores the relationship between the psychological feelings of people with visual impairment and sound environment, urban space, reflecting a certain research depth. At the same time, in terms of research methods, the research chain of “visual impaired people—urban space” is constructed through sound environment evaluation, which can provide reference for relevant research.

This study has some limitations. Due to the particularity of the visually impaired participants, only 24 volunteers were recruited during the research phase, and it was difficult to recruit more volunteers in a short period of time. Therefore, such a sample size may have impacted the research conclusions. Secondly, due to the limitations of the experimental site conditions and the participants’ own requirements, the single point sound source playback mode was used to let the participants participate concurrently to reduce the total time of the experiment. This may have caused a loss of some sound signals, such as the spatial position information of a sound source, which may have impacted the research conclusions. Therefore, future research should increase the number of volunteers for visually impaired people and improve the experimental location and equipment conditions. Moreover, comparative experiments with people who are not visually impaired should be conducted to explore the evaluation characteristics of urban spaces by different groups of people; thus, enriching the overall research conclusions.

## Conclusion

In this study, the visually impaired population underwent a five-dimensional psychological perception evaluation of seven types of urban space sound environments in Tianjin City. The results of the non-parametric test showed that there were significant differences in the evaluations of three dimensions: comfort, safety, and clarity. Among them, the overall evaluation of parks, residential areas, and shopping streets was positive, while the evaluation of bus stops, hospitals, markets, and urban departments was negative.

In the results of the analysis, the types of spaces with a positive impact had the following characteristics: lower equivalent sound pressure levels, with decibel levels between 40 and 60 dB, and the types of sound were mainly lower traffic, chat, and hawker sounds. These scenes were far from traffic roads or had low traffic road levels, a small number of vehicles and people, and were mainly living, leisure, and commercial urban spaces belonging to life-type urban spaces. The types of spaces with a negative impact were characterized by high equivalent sound levels, with decibel levels between 60 and 70 dB. The main types of sounds included horn, manhole cover, construction, and higher traffic sounds. These scenes were adjacent to urban roads, characterized by many people and vehicles, had traffic, and belonged to complex-type urban spaces.

Thus, visually impaired people feel anxious and fearful due to traffic, horn, and construction sounds. They are prone to feel helpless when crowded with mixed function spaces or close to main urban roads. This suggests that visually impaired people want a quieter and purer urban space, which is helpful for them to make accurate judgments about the external environment. The characteristics of this type of space should be far away from the main roads with a large flow of people, composed of street spaces dominated by living and leisure functions, and include a moderate density of people. Therefore, when designing travel places, barrier-free facilities and travel routes for visually impaired people should be included in urban construction and planning. This will enable visually impaired people to conduct daily activities more smoothly and, ultimately, achieve the overall goal of smart and fair urban development. Overall, this study compensated for a lack of research in related fields in China. Moreover, it also enriched the basic database of visually impaired individuals in China and provided references for the design of urban spaces, activity centers, and barrier-free systems for visually impaired individuals.

## Data Availability Statement

The original contributions presented in the study are included in the article/supplementary material, further inquiries can be directed to the corresponding author/s.

## Ethics Statement

The studies involving human participants were reviewed and approved by the Tianjin Disabled Persons’ Federation and Tianjin Association of the Blind. The patients/participants provided their written informed consent to participate in this study.

## Author Contributions

SZ, KZ, MZ, and XL: conceptualization and methodology. KZ and MZ: software. XL: validation, data curation, writing—review, and editing. KZ, MZ, and XL: formal analysis. SZ, KZ, and MZ: investigation and writing—original draft preparation. SZ and XL: supervision. SZ: funding acquisition. All authors contributed to the article and approved the submitted version.

## Conflict of Interest

The authors declare that the research was conducted in the absence of any commercial or financial relationships that could be construed as a potential conflict of interest.

## Publisher’s Note

All claims expressed in this article are solely those of the authors and do not necessarily represent those of their affiliated organizations, or those of the publisher, the editors and the reviewers. Any product that may be evaluated in this article, or claim that may be made by its manufacturer, is not guaranteed or endorsed by the publisher.
